# Leishmaniasis Diagnosis via Metagenomic Next-Generation Sequencing

**DOI:** 10.3389/fcimb.2020.528884

**Published:** 2020-09-23

**Authors:** Hongbin Chen, Chunhong Fan, Hua Gao, Yuyao Yin, Xiaojuan Wang, Yawei Zhang, Hui Wang

**Affiliations:** Department of Clinical Laboratory, Peking University People's Hospital, Beijing, China

**Keywords:** leishmaniasis, *Leishmania*, mNGS, diagnosis, infection

## Abstract

Leishmaniasis is a vector-borne disease caused by *Leishmania*. Although the incidence of leishmaniasis in China is currently low, it has not been completely eradicated. In 2019, visceral leishmaniasis was diagnosed in three patients using bone marrow microscopic examination and metagenomic next-generation sequencing (mNGS). The bone marrow mNGS results from the three patients indicated that 99.9, 99.6, and 30.3% of non-human reads matched the *Leishmania* genome, and plasma mNGS results from one of the patients revealed that 46.2% of non-human reads matched the *Leishmania* genome. In the second patient's plasma, no *Leishmania* sequences were detected by plasma mNGS, and the third patient's plasma was unavailable. The pathogen in all three patients was identified as *Leishmania infantum*. *Leishmania* amastigotes were observed by microscopic examination of bone marrow smears in all three patients, but were not found in peripheral blood smears. This indicates that the sensitivity of mNGS is higher than that of smear microscopy and that mNGS can be used to identify *Leishmania* at the species level. All three patients were elderly male farmers, two from Shanxi and one from Beijing. All three patients had splenomegaly and pancytopenia. Originally, these patients were misdiagnosed and treated for extended periods in other hospitals. Diagnoses of visceral leishmaniasis took place 6, 2, and 2 months after the onset of symptoms in the three patients. In conclusion, this study confirms that bone marrow mNGS can be used to quickly and accurately confirm a diagnosis in patients with suspected leishmaniasis.

## Introduction

Leishmaniasis is a zoonotic disease caused by *Leishmania* that is transmitted between arthropods and mammals. It is endemic to large areas of tropical, subtropical, and Mediterranean basins, and it is found in 98 countries (Alvar et al., 2012). Each year, there are ~0.2–0.4 million cases of visceral leishmaniasis, 0.7–1.2 million cases of cutaneous leishmaniasis and ~20,000–40,000 leishmaniasis-related deaths (Alvar et al., 2012). Therefore, leishmaniasis is a global public health problem. Visceral leishmaniasis has existed in China for at least 120 years (Lun et al., 2015). In 2000, the incidence of visceral leishmaniasis in Xinjiang, Gansu, and Sichuan, China was ~1, ~0.5, and ~0.7 per 100,000, respectively (Lun et al., 2015). In 2011, the incidence of visceral leishmaniasis was 0.03 per 100,000 in China (Lun et al., 2015). From 2002 to 2011, a total of 3,169 visceral leishmaniasis cases were reported throughout China, with approximately 140 to 509 cases per year (Lun et al., 2015). This suggests that leishmaniasis has not been eradicated in China and remains a public health problem.

Currently, methods for detecting *Leishmania* infection include microscopy (Carneiro et al., 2017), *in vitro* culturing (Ates et al., 2013), isolation of experimental animals (Loria-Cervera and Andrade-Narvaez, 2014), dermal diagnostic tests (Weigle et al., 1991), xenodiagnosis (Sadlova et al., 2015), and molecular approaches (Galluzzi et al., 2018; Sundar and Singh, 2018; Conter et al., 2019). Each method has its own advantages and disadvantages. Although microscopic examination is simple and cost-effective, its sensitivity depends on the number of parasites and their distribution in the sample, the sampling process, and the technical skills of personnel, and it cannot distinguish between *Leishmania* species. *In vitro* culturing and isolation of experimental animals is time-consuming and expensive. Although the dermal diagnostic test (Weigle et al., 1991) is simple, sensitive, and specific, it cannot distinguish between *Leishmania* species or between past and current infections. Similarly, although xenodiagnosis (Sadlova et al., 2015) has high specificity and reasonable sensitivity, it is time-consuming and cannot distinguish between *Leishmania* species. In relative terms, molecular methods have the advantage of being fast, sensitive, and specific; however, these methods still present problems with false positives and false negatives.

As next-generation sequencing (NGS) technology has developed, metagenomic NGS (mNGS) has begun to be applied to the etiological diagnosis of infectious diseases (Wilson et al., 2014; Thoendel et al., 2018; Blauwkamp et al., 2019), including parasite infections (Kounosu et al., 2019). mNGS can be used to directly identify potential pathogens such as bacteria, fungi, viruses, and parasites in DNA samples by high-throughput sequencing and database comparison without the need to isolate pathogens. Compared with traditional pathogen detection methods, mNGS provides obvious advantages for the identification of pathogens that cannot be cultured or are not easily cultured. In this study, mNGS was used to identify *Leishmania* in the bone marrow of three visceral leishmaniasis patients, demonstrating its important role in clinical diagnosis and treatment. To our knowledge, this is the first report of leishmaniasis diagnosis by mNGS in China.

## Materials and Methods

This study was approved by Peking University People's Hospital Institutional Review Board (ID: 2019PHB134).

Beginning on February 11, 2019, we performed traditional microbiological testing and in-house mNGS on specimens from patients with fever of unknown origin and suspected infection. In addition, we collected patients' demographic information, clinical symptoms, laboratory test results, imaging examination results, diagnosis and treatment history, and prognosis results. Through this process, we confirmed three cases of visceral leishmaniasis using mNGS combined with microscopic examination.

### mNGS Protocol

A QIAamp DNA Microbiome Kit (Qiagen) was used for DNA extraction from bone marrow and plasma. Sterile deionized water was extracted alongside the test specimens as an external negative control. DNA concentrations were determined using the Qubit dsDNA HS (High Sensitivity) Assay Kit (Thermo Fisher Scientific). Single-end library were prepared using a Vazyme TruePrepTM DNA Library Prep Kit (Vazyme). Sequencing was performed using a NextSeq 550 System (Illumina). Clean reads obtained after adapter trimming and low-quality and low-complexity filtering underwent microbial identification. One human (hg38), 185,630 bacterial, 308 archaeal, 54 fungal, 9,021 viral, 178 invertebrate, and 39 protozoan reference genomes were downloaded from NCBI, and reference databases were created using Kraken2. Taxonomy was determined using Kraken2 v2.0.8-beta (Wood and Salzberg, 2014) and bacterial, archaeal, fungal, viral, and protozoan databases from May 2019. Approximately 20 million read pairs were generated for each sample. The sequenced data have been submitted to the Short Read Archive under Bioproject: PRJNA650011.

## Results

Patients who were suspected to have leishmaniasis by their clinicians were assessed by bone marrow microscopy and mNGS analysis. We found a total of three patients with visceral leishmaniasis. These patients were elderly men who had fever, cough, sputum production, fatigue, and anorexia (Table 1). The patients' detailed medical records are described below.

**Table 1:** Clinical characteristics of patients.

**Patient No./Sex/Age, year**	**Final diagnosis**	**Clinical presentation**	**Disease duration at time of diagnostic, mo**
No. 1/Male/53	Visceral leishmaniasis	Splenomegaly, pancytopenia, fever, night sweats, epistaxis, cough, expectoration	6
No. 2/Male/80	Visceral leishmaniasis	Splenomegaly, pancytopenia, cough, expectoration, fatigue, anorexia	2
No. 3/Male/65	Visceral leishmaniasis	Splenomegaly, pancytopenia, anorexia, bloating, fever, fatigue	2

### Case Descriptions

**Figure 1:**
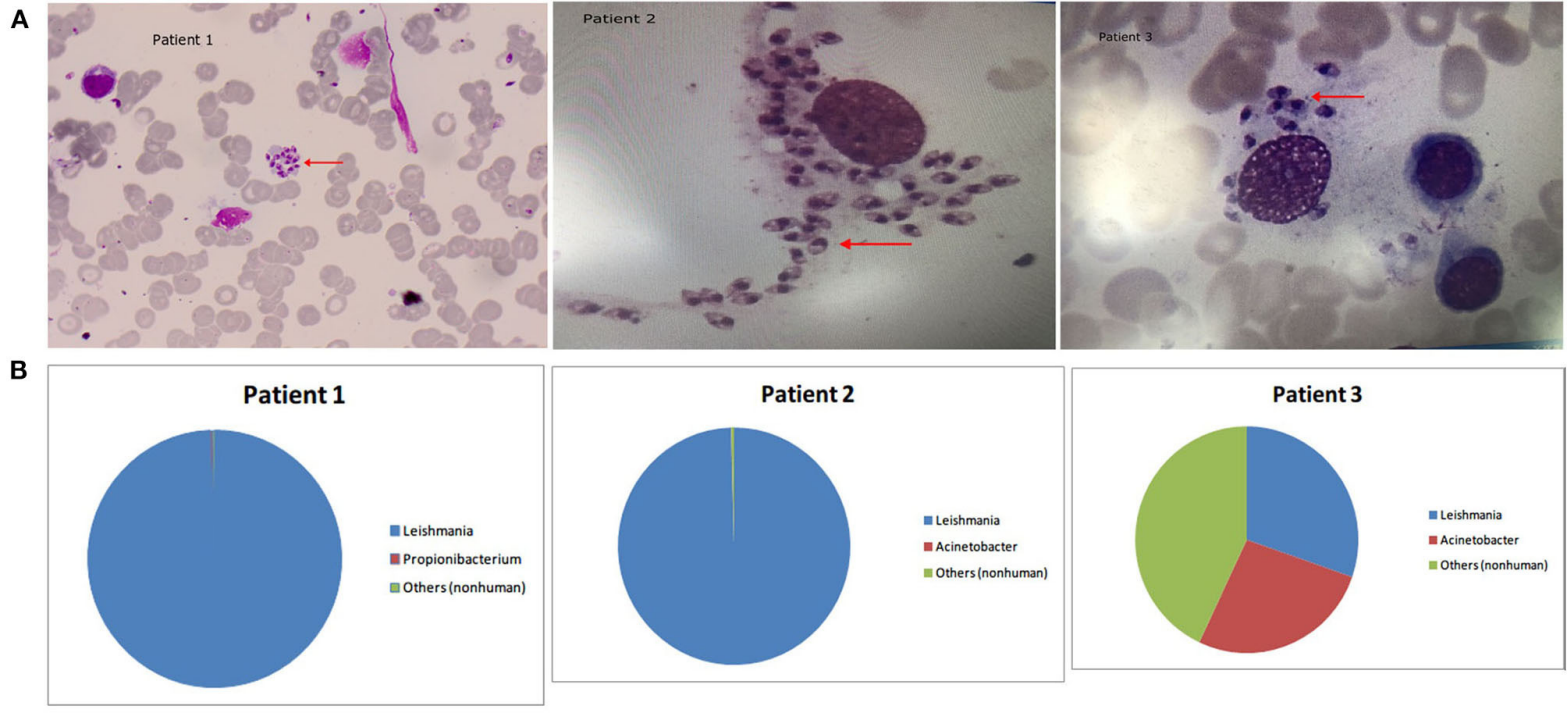
**(A)** Arrowheads show *Leishmania* amastigotes, which are oval and 2.9–5.7 × 1.8–4.0 μm in size. The cytoplasm is stained lilac or purple-blue by Wright's stain and contains a large round nucleus. The nucleus (red or lavender) is located at the front of the worm and accounts for a third to a half of the worm's length. The moving matrix is located next to the nucleus and is rod-shaped and dark in color. **(B)** The distribution of non-human sequences identified in patients' bone marrow.

**Patient 1** was a 53-year-old male farmer from Shanxi province, China who was admitted to hospital for “intermittent fever for 6 months and sustained fever for more than 1 month.” The patient had night sweats, chills, epistaxis, cough, and yellow sputum production for 1 month, as well as recurrent parotid enlargement. The following abnormal laboratory test results were obtained: white blood cell count, 3.7 × 10^9^ cells/L; neutrophil percentage, 18.1%; lymphocyte percentage, 73.60%; hemoglobin, 80 g/L; serum glutamic-oxaloacetic transaminase, 48 U/L; total protein, 89.2 g/L; albumin, 27.6 g/L; C-reactive protein, 30 mg/L; anti-nuclear antibody titer, 1:80; anti-dsDNA antibodies, 54.9 IU/mL; anti-histone antibody, 64.08 RU/mL; and rheumatoid factor, 825 IU/mL. Abdominal color Doppler ultrasound indicated enlarged liver, slightly widened portal veins, fatty liver, and splenomegaly. In the 6 months prior to arriving at our hospital, this patient was diagnosed with “hypoproteinemia, hyperglobulinemia, hemolytic anemia, systemic lupus erythematosus, secondary Sjogren's syndrome (not excluded), lymph tumors (not excluded), and autoimmune hemolytic anemia.” The patient's condition did not improve after 6 months of treatment due to incorrect diagnosis. After this patient visited our hospital, his bone marrow was tested by microscopy and mNGS. Wright's staining of the bone marrow revealed *Leishmania* amastigotes (Figure 1). After removing human reads, a total of 1,221,754 reads were produced by mNGS, of which 99.9% (1,220,942/1,221,754) mapped to the *Leishmania* genome (Table 2). We also performed mNGS on the patient's plasma and produced a total of 1,559 non-human reads, of which 46.2% (721/1559) mapped to the *Leishmania* genome (Table 2). Bone marrow and plasma mNGS results showed that 6,915 and seven reads aligned with the *Leishmania infantum* genome, respectively. After the patient was diagnosed with visceral leishmaniasis, he was given antimony therapy. The patient was discharged from the hospital when hemoptysis occurred on the third day of treatment.

**Table 2:** Summary of metagenomic sequencing results of patients.

**Patient no**.	**Specimen type**	**Pathogen**	**Unique non-human non-redundant reads, no**.	**Unique pathogen reads, no (% non-human reads)**
1	Bone marrow	*Leishmania*	1,221,754	1,220,942 (99.9)
1	Plasma	*Leishmania*	1,559	721 (46.2)
2	Bone marrow	*Leishmania*	18,150	18,070 (99.6)
2	Plasma	No pathogen	34	0 (0)
3	Bone marrow	*Leishmania*	1445	438 (30.3)

**Patient 2** was an 80-year-old male farmer from Beijing. He had been experiencing cough, expectoration, fatigue, and anorexia for 2 months. The abnormal laboratory test results included the following: white blood cell count, 1.08 × 10^9^ cells/L; monocyte percentage, 13.9%; hemoglobin content, 107 g/L; aspartate aminotransferase, 52 U/L, γ-glutamyl transpeptidase, 103 U/L; alkaline phosphatase, 207 U/L; creatine kinase, 26 U/L; total protein, 94.8 g/L; albumin, 30 g/L; albumin/globulin, 0.46; immunoglobulin G, 47.3 g/L; immunoglobulin M, 6.22 g/L; complement C3, 0.427 g/L; complement C4, 0.114 g/L; rheumatoid factor, 1,990 IU/mL; and serum ferritin, 1,335.21 ng/mL. The patient had previously been diagnosed with hypertension and diabetes. At the local hospital, the patient was diagnosed with bronchitis and treated using levofloxacin, azithromycin, ambroxol, vitamin B4, and recombinant human granulocyte colony stimulating factor for 2 months, but his condition did not improve and he was transferred to our hospital. In our hospital, the results of bone marrow mNGS tests showed that almost all non-human sequences (18,070/18,150) belonged to *Leishmania* (Table 2 and Figure 1), and further analysis revealed that they were *L. infantum* sequences. Nevertheless, no pathogen sequences were detected by plasma mNGS. The bone marrow contained a small number of nucleated cells, and *Leishmania* amastigotes were found (Figure 1). Sodium antimonate gluconate was injected for 13 days. The treatment was interrupted due to nose bleeding, and the patient was discharged from hospital with recommendations to repeat the treatment in 1 month.

**Patient 3** was a 65-year-old male farmer from Shanxi. The patient had not experienced obvious anorexia, abdominal distention, or increased exhaustion accompanied by fatigue and lethargy for 2 months. There was no obvious cause of fever for the first 3 weeks, and the patient's body temperature peaked at 39°C. The patient's body temperature dropped after administration of nimesulide, whereas fever recurred after withdrawal for 1–2 days. Physical examination revealed enlargement of the spleen to 2 cm under the ribs, liver dullness, and mobile dullness. The following abnormal laboratory test results were obtained: leukocyte count, 1.76 × 10^9^ cells/L; absolute neutrophil count, 1.04 × 10^9^ cells/L; hemoglobin content, 99 g/L; platelet count, 33 × 10^9^ cells/L; alanine aminotransferase, 202 U/L; aspartate aminotransferase, 287 U/L; gamma glutamyl transpeptidase, 71 U/L; alkaline phosphatase, 154 U/L; lactate dehydrogenase, 523 U/L; alpha hydroxybutyrate dehydrogenase, 349 U/L; albumin, 22.5 g/L; total cholesterol, 2.28 mmol/L, high-density lipoprotein cholesterol, 0.31 mmol/L; low-density lipoprotein cholesterol, 1.11 mmol/L; total bilirubin, 77.9 μmol/L; direct bilirubin, 58.5 μmol/L; calcium, 2.12 mmol/L; albumin/globulin, 0.45; potassium, 5.33 mmol/L; sodium, 133.0 mmol/L; prothrombin time, 15.1 s; prothrombin activity, 62%; international standardized ratio of prothrombin, 1.40; fibrinogen, 106 mg/dL; activated partial thromboplastin time, 37.4 s; fibrin degradation products, 40.2 μg/mL; D-dimer, 5,008 ng/mL; C-reactive protein, 84.72 mg/L; procalcitonin 1.57 μg/L; ferritin, 4,190 ng/mL; folic acid, 3.27 ng/mL; blood kappa light chain, 19.90 mg/dL; urine lambda light chain, 1,920 mg/dL; urine kappa light chain, 3,040 mg/dL; immunoglobulin G, 38.2g/L; complement C3, 0.426 g/L; complement C4, 0.115 g/L; and rheumatoid factor, 44.0 IU/mL. Ultrasound of the abdomen indicated liver cirrhosis, splenomegaly, parasplenic lesions, and a small number of ascites. Computerized tomography scan of the thorax, abdomen, and pelvic cavity indicated bilateral pleural effusion, emphysema scattered in both lungs, slightly enlarged mediastinal lymph nodes, liver cirrhosis, portal hypertension, splenomegaly, spleen varices, abdominal pelvic effusion, thickening of the Glisson sheath, gallbladder wall thickening and edema, and partial intestinal wall thickening and edema. For 2 months, the patient was misdiagnosed and treated at the local hospital, but his condition did not improve. He was subsequently referred to our hospital, where the results of bone marrow mNGS showed that *Leishmania* sequences accounted for 30.3% of non-human reads (438/1,445) (Table 2), ranking first among the detected pathogens (Figure 1). Further analysis identified the sequences as *L. infantum*. Bone marrow smears indicated active bone marrow hyperplasia, reduced platelet numbers, and *Leishmania* amastigotes (Figure 1). After the patient was admitted to the hospital, his fever recurred, and meropenem combined with moxifloxacin was used to treat the infection. The patient was also treated using compound glycyrrhizin, polyene phosphatidylcholine, albumin, and platelet transfusion. The patient had a clear diagnosis of hemophagocytic syndrome and was given dexamethasone (15 mg qd) and gamma globulin (20 g qd). The patient's liver function improved, but fever still recurred. He was transferred to a specialist hospital for treatment of visceral leishmaniasis after *Leishmania* were identified.

## Discussion

The current passive surveillance system for visceral leishmaniasis in China reported a total of 2,450 cases between 2005 and 2010, with an average of 408 cases per year (Wang et al., 2012). Of these, 97.71% of cases occurred in Xinjiang, Gansu, and Sichuan provinces. In the present study, two of the patients with visceral leishmaniasis were from rural Shanxi and one was from rural Beijing. Shanxi is still an epidemic area for visceral leishmaniasis, whereas Beijing has not been considered an epidemic area since the 1950s (Lun et al., 2015). The present study represents the first documented case in Beijing since the 1950s, which is cause for alarm. In China, visceral leishmaniasis is divided into three types according to the epidemiological characteristics of the disease: the anthroponotic type (AVL), the zoonotic mountain type (MT-ZVL), and the zoonotic desert type (DT-ZVL) (Lun et al., 2015). MT- ZVL occurs in mountainous areas in the west and hilly areas in Gansu, Sichuan, Shaanxi, and Shanxi (Wang et al., 2012). *Leishmania infantum* is usually the cause of MT-ZVL. Infection rates of *L. infantum* are high in dogs (Wang et al., 2011), and dogs are reservoirs of *L. infantum*. Therefore, because the pathogens identified in the three cases in this study were *L. infantum*, the infection source for these patients may have been dogs.

The standard criteria for diagnosis of visceral leishmaniasis include clinical symptoms, history of living or traveling to epidemic areas, and positive laboratory tests. Splenomegaly is the most common clinical manifestation of visceral leishmaniasis. Therefore, a history of traveling to an epidemic area and splenomegaly can be used to make a preliminary diagnosis. In the present study, splenomegaly occurred in all three patients, and two patients came from an epidemic area, but one patient had no history of living in or traveling to an epidemic area. Other typical clinical manifestations include pancytopenia, which was also observed in the three patients in this study. Other atypical clinical symptoms include irregular fever, fatigue, lethargy, and weight loss. These clinical symptoms were observed to varying degrees in the three patients in this study. The gold standard for the diagnosis of visceral leishmaniasis is microscopic visualization of *Leishmania* amastigotes in specimens or cultured specimens. However, because there are currently few professionals trained in parasite morphology in most clinical laboratories in China, the rate of false negatives by microscopic examination is very high. A previous study found that, of 1,093 confirmed cases of visceral leishmaniasis in China, only eight cases were identified by microscopic examination (Fu et al., 2013). In order to avoid the high incidence of false negatives by microscopic examination due to a lack of personnel, many clinical laboratories in China diagnose leishmaniasis by detection of *Leishmania* antibodies or antigens (Gao et al., 2015; Jiang et al., 2016). In recent years, the detection of *Leishmania*-specific genes by polymerase chain reaction has been carried out in clinical laboratories (Zhao et al., 2015), but its use is far from universal. Here, we present three diagnostically challenging cases of visceral leishmaniasis in which *Leishmania* was identified by the combined use of mNGS and microscopic examination of bone marrow. The results of this study showed that many *Leishmania* sequences were detected in the bone marrow of the three patients diagnosed with visceral leishmaniasis. *Leishmania* amastigotes were also identified in all three cases. In clinical laboratories that lack the ability to identify parasites by morphological examination, mNGS is undoubtedly a very useful tool to diagnose visceral leishmaniasis. However, *Leishmania* sequences were detected in plasma in only one of the three cases of visceral leishmaniasis in this study. Therefore, using only plasma mNGS may prevent detection of *Leishmania*. In contrast, bone marrow mNGS is a good choice for *Leishmania* detection. In addition to *Leishmania*, many other pathogens, such as *Brucella* (Tuon et al., 2017), *Salmonella* (Crump et al., 2015), *Mycobacterium tuberculosis* (Wang et al., 2018), *Histoplasma capsulatum* (Medina-Pinon et al., 2017), and *Penicillium marneffei* (Qin et al., 2015), can infect the bone marrow. When pathogens are unknown, high-throughput mNGS can be used for identification. This demonstrates that bone marrow mNGS is a powerful tool to identify rare pathogens causing bone marrow infections.

In summary, this study reports the first case of visceral leishmaniasis in Beijing since the 1950s, which suggests that infections caused by rare pathogens cannot be ignored in non-endemic areas. Three cases of visceral leishmaniasis were diagnosed by mNGS combined with bone marrow smears, which indicates that mNGS can be used to assist in the diagnosis of infections caused by difficult-to-culture pathogens.

## Data Availability Statement

All datasets generated for this study are included in the article/supplementary material.

## Ethics Statement

This study was approved by the Research Ethics Board at Peking University People's Hospital (ID: 2019PHB134).

## Author Contributions

HC and HG performed bioinformatics analysis. HC participated in the writing of the manuscript. CF collected cases. YY, XW, and YZ performed mNGS test. HW conceived the project and participated in the writing of the manuscript. All authors read and approved the final manuscript.

## Conflict of Interest

The authors declare that the research was conducted in the absence of any commercial or financial relationships that could be construed as a potential conflict of interest.
